# A Comparative Peptidomic Characterization of Cultured Skeletal Muscle Tissues Derived From *db/db* Mice

**DOI:** 10.3389/fendo.2019.00741

**Published:** 2019-10-29

**Authors:** Yanting Wu, Mei Han, Yan Wang, Yao Gao, Xianwei Cui, Pengfei Xu, Chenbo Ji, Tianying Zhong, Lianghui You, Yu Zeng

**Affiliations:** ^1^Nanjing Maternity and Child Health Care Institute, Women's Hospital of Nanjing Medical University (Nanjing Maternity and Child Health Care Hospital), Nanjing, China; ^2^Department of Clinical Laboratory, Women's Hospital of Nanjing Medical University (Nanjing Maternity and Child Health Care Hospital), Nanjing, China; ^3^Affiliated Maternity and Child Health Care Hospital of Nantong University, NanTong, China; ^4^Department of Endocrinology, Children's Hospital of Nanjing Medical University, Nanjing, China

**Keywords:** mass spectrometry, secreted peptide, skeletal muscle, insulin resistance, Irs1-Akt signaling pathway, Pgc1α

## Abstract

As an important secretory organ, skeletal muscle has drawn attention as a potential target tissue for type 2 diabetic mellitus (T2DM). Recent peptidomics approaches have been applied to identify secreted peptides with potential bioactive. However, comprehensive analysis of the secreted peptides from skeletal muscle tissues of *db/db* mice and elucidation of their possible roles in insulin resistance remains poorly characterized. Here, we adopted a label-free discovery using liquid chromatography tandem mass spectrometry (LC-MS/MS) technology and identified 63 peptides (42 up-regulated peptides and 21 down-regulated peptides) differentially secreted from cultured skeletal muscle tissues of *db/db* mice. Analysis of relative molecular mass (Mr), isoelectric point (pI) and distribution of Mr vs pI of differentially secreted peptides presented the general feature. Furthermore, Gene ontology (GO) and pathway analyses for the parent proteins made a comprehensive functional assessment of these differential peptides, indicating the enrichment in glycolysis/gluconeogenesis and striated muscle contraction processes. Intercellular location analysis pointed out most precursor proteins of peptides were cytoplasmic or cytoskeletal. Additionally, cleavage site analysis revealed that Lysine (N-terminal)-Alanine (C-terminal) and Lysine (N-terminal)-Leucine (C-terminal) represents the preferred cleavage sites for identified peptides and proceeding peptides respectively. Mapped to the precursors' sequences, most identified peptides were observed cleaved from creatine kinase m-type (KCRM) and fructose-bisphosphate aldolase A (Aldo A). Based on UniProt and Pfam database for specific domain structure or motif, 44 peptides out of total were positioned in the functional motif or domain from their parent proteins. Using C2C12 myotubes as cell model *in vitro*, we found several candidate peptides displayed promotive or inhibitory effects on insulin and mitochondrial-related pathways by an autocrine manner. Taken together, this study will encourage us to investigate the biologic functions and the potential regulatory mechanism of these secreted peptides from skeletal muscle tissues, thus representing a promising strategy to treat insulin resistance as well as the associated metabolic disorders.

## Introduction

Skeletal muscle is considered as the primary tissue for insulin-stimulated glucose uptake, accounting for up to 80% of the insulin-dependent glucose disposal in whole body glucose homeostasis (1). Accordingly, the dysregulation of skeletal muscle metabolism also arise a number of metabolic disorders such as hyperinsulinaemia, excessive hepatic gluconeogenesis (2), abnormal lipid accumulation (3), impaired glucose uptake and metabolic inflexibility (4). Furthermore, disorders in skeletal muscle play a central role in the development of type 2 diabetic mellitus (T2DM), obesity and lead to other related complications (5). Thus, it is of great interest to deeply characterize the pathogenesis of dysregulation on skeletal muscle glucose/lipid homeostasis to whole-body endocrine and metabolic functions.

As an important secretory organ, skeletal muscle has drawn attention to be a potential target tissue for treating metabolic disorders (6). Thus, analysis of skeletal muscle secretome opens up a novel route for comprehending the communication of this tissue with other tissues such as adipose tissue (7), bone (8), liver (9) and pancreas (10, 11). In light of recently reported experimental evidence, a variety of proteins generated by muscles fibers and released into the circulation are classified as myokines (12), most of which have autocrine, paracrine, and endocrine effects not only in muscle fiber growth (13) but also systemic metabolism (14). The first identified myokine IL-6 presented a vital locally muscular effects such as skeletal muscle growth and glucose/lipid metabolism (15, 16). Furthermore, IL-6 also could be released from contracting muscle, exerting endocrine effects on peripherally insulin sensitive tissues (17–19). Another known contraction-induced myokines including IL-15, IL-8, Irisin, and Myonectin showed potential metabolic function for preventing and treating T2DM (20). These accumulating evidence of myokine from skeletal muscle secretome are central to our understanding of the cross talk between skeletal muscle and other organs during exercise. However, knowledge of the skeletal muscle secretome is scarcely reported under pathophysiology of metabolic diseases such as T2DM and obesity. Identification of more types of muscle-secreted factors and exploration of the potential regulatory mechanisms by which they act remain to be established.

Peptides in length of 3–50 amino acids residues, which are widely characterized in mouse and human, are termed as a sort of compounds produced or secreted by endocrine gland tissues as well as certain types of cells (21, 22). And these endogenous peptides have important physiological action, including neuroregulation (23), cell differentiation (24) and energy metabolism (25) and dysregulation of peptide hormone signaling have been implicated in a wide range of diseases (26, 27). In view of that, further insight into identification of novel peptides is of major importance. Benefited from the progresses in peptide extraction method and application of modern analytical methods, (U)HPLC, nano-LC and CE, hyphenated with tandem mass spectrometry (MS/MS) technology (28, 29), various techniques used for quantitative peptidomics have been applied to address the challenging question of identifying peptides with potential bioactive under the physiological or disease condition (30). More importantly, the peptidomics is widely used to identify biological markers (31), discover new drug (32) and therapeutic targets (33). Recently, quantative peptidomics has also been conducted in endocrine studies (22, 34, 35), however, the secreted peptidomics from skeletal muscle under the insulin-resistant condition was not fully characterized.

Herein, we performed liquid chromatography tandem mass spectrometry (LC-MS/MS) technology to help characterize the secretome from cultured skeletal muscle tissues of *db/db* mice at peptides level and identify putative bioactive peptides. A global secreted peptides were established and bioinformatics analysis of precursor proteins provided a possible relationship of differential peptides with T2DM or insulin resistance. Additionally, the biological effects of these secreted peptides on C2C12 myotubes elucidated a possible regulatory role in insulin signaling- and mitochondrial-related genes expression. Taken together, these observations will encourage us to investigate function of these secreted peptides from cultured skeletal muscle tissues with other tissues under the diabetic state, thus representing a promising strategy for prevention and treatment of insulin resistance as well as the associated metabolic disorders.

## Materials and Methods

### Ethics Statement

All the studies involving mice acquired approval from the Ethical Committee of Nanjing Medical University. All procedure involving mice were carried out in accordance with the guidelines of the Institutional Animal Care and Use Committee of Nanjing Medical University (Approval Number: IACUC-1812053).

### Animal Experiments and Sample Preparation

Twelve-week-old male C57BLKS/J *db/db* mice (*n* = 8, *db/db* group) and age-matched WT controls (*n* = 8, NC group) were purchased from the Model Animal Research Center of Nanjing Medical University. After adaptive raising for one week, mice were sacrificed by cervical dislocation and skeletal muscle tissues were isolated from the left hind leg (each mice of 100 ~ 150 mg). Subsequent operations were carried out under a laminar airflow hood to decrease contamination. The visible blood vessels and connective tissue were removed from the tissue. After rinsed with PBS, the skeletal muscle tissues were cut into small pieces (3–4 mm^3^) with scissors as described by an established protocol (36, 37). Tissue cutting will lead to release of damaged cells slowly into the medium. Additionally, a small amount of serum proteins in the tissue pieces will diffuse out during culture period. Therefore, necessary washing procedures during culture were adopted to obtain medium samples (referred to as secretome) containing mainly skeletal muscle tissue-derived secreted components as previously reported (38, 39). Tissue fragments were placed in a 10 cm plate (200~300 mg from two mice as one sample) containing 10 mL serum/phenol red free DMEM/F12 medium (Gibco, Grand Island, CA, USA). After incubation in a humidified incubator at 37°C under 95% O2 and 5% CO2 for 48 h, the medium was immediately supplemented with protease inhibitors and centrifuged (845 g, 10 min, 4°C) to wipe off cell debris and dead cell. Supernatant samples from each group (NC or *db/db* group) were harvested from four individual tissue culture dishes independently (*n* = 4 per group). Lactate dehydrogenase (LDH) (36, 40, 41) and IL-6 (42) expression levels were detected to evaluate skeletal muscle vitality and capability during the *in vitro* culture. Then the supernatant samples obtained were stored at −80°C until further processing.

### Peptide Extraction and Desalting

First, both supernatant samples were concentrated to 1–2 mL by centrifuges for speed vacuum (LaboGene, Allerød, Denmark). Then equal volume of U2 solution containing 8 M urea and 100 mM tetraethyl-ammonium bromide in pH 8.0 was added to the concentrated supernatant for denaturation. Followed by centrifugation for 30 min (13,000 g, 30°C), the medium supernatant was transferred to a new centrifuge tube. Subsequently, proteins were reduced by 10 mM dithiothreitol and alkylated with 55 mM iodoacetamide successively. The protein concentrations of supernatant from cultured skeletal muscle samples were detected by Bradford method (43) and integrity of these samples were evaluated by sodium dodecyl sulfate (SDS)-polyacrylamide gel electrophoresis (PAGE) combined with silver staining. Afterwards the peptides were separated from samples by treatment with Amicon^®^ Ultra Centrifugal Filters in 10-kDa (Merck Millipore, Billerica, MA, USA) according to the manufacturer's instruction as previously described (37). The “peptidome” present in the filtrates was desalted using a Strata X C18 column (Phenomenex, Torrance, CA, USA) and the desalted peptide solution was vacuum-dried with centrifuges for speed vacuum (SCAN SPEED 40, LaboGene) as previously described (44) and immediately frozen at −80°C until the following processing.

### Liquid Chromatography Tandem Mass Spectrometry (LC-MS/MS)

The LC-MS/MS analysis was conducted similarly to the previous protocols (45). The peptide samples were redissolved in 2% (v/v) acetonitrile/0.1% formic acid (FA) (v/v) and 5 μL solution was injected into an A Triple TOFTM 5600 mass spectrometer (AB Sciex, Redwood City, CA, USA) coupled to a ekspert™ nanoLC400 liquid chromatography (AB Sciex) via a nanosource electrospray interface equipped with distal coated SilicaTip emitters (New Objective, Woburn, MA, USA). First, load peptide onto a C18 trap column (5 μm, 100 μm ×20 mm, AB Sciex) and elute at 300 nL/min onto a C18 analytical column (3 μm, 75 μm ×150 mm, Welch Materials, Shanghai, China) in the gradient as long as 120 min. These two mobile phases included buffer A containing 2% acetonitrile/ 0.1% FA/ 98% H2O (v/v) and buffer B containing 98% acetonitrile/0.1% FA/2% H2O (v/v). Then peptide mixture was eluted at a flow rate of 0.3 μL/min in a gradient generated with Solvent A containing 98% water and 2% acetonitrile containing 0.1% FA (v/v) and Solvent B containing 2% water and 98% acetonitrile containing 0.1% FA (v/v) according to the previously described reports (45, 46). The mass spectrometer was operated in positive mode with a spray voltage of 2500 V, 206.84kPa for the curtain gas, 41.37kPa for the nebulizer gas and 150°C as temperature. A data dependent acquisition (DDA) method was applied and a full scan MS spectrum (300–1500 m/z) with accumulation time of 0.25 s was adopted. Top 30 precursor ions for fragmentation based on the highest intensity were selected. The collections of MS1 spectra were in the range 350–1500 m/z, and MS2 spectra were in the range of 100–1500 m/z. A total of 47,709 MS/MS were collected from all LC-MS/MS analyses.

### Peptide Identification and Quantitative Analysis

Protein Pilot Software (https://sciex.com.cn/products/software/proteinpilot-software, version4.5,AB SCIEX) was adopted to analyze the original MS/MS file data (47). For peptides identification, the Paragon algorithm was employed against the Mus_musculus SwissProt sequence database (a total of 85210 items, updated in January 2019). The following parameters were installed: The parameters were set as follows: 1) cysteine modified with iodoacetamide; 2) biological modifications were selected as ID focus. The strategy of the automatic decoy database search was employed to estimate false discovery rate (FDR) calculation using the Proteomics System Performance Evaluation Pipeline (PSPEP) Software integrated in protein pilot-software. Only unique peptides (global FDR values <1%) were considered for further analysis. Skyline v4.2 software was employed for MS1 filtering and ion chromatogram extractions for peptides label-free quantification (48, 49). And the parameters setting for skyline MS1 filtering were according to previous studies (48). Using the results of the Skyline quantification, the mean value of the ratio of each group was used to calculate the fold change.

### Bioinformatics and Annotations

The relative molecular mass and isoelectric point of each peptide were calculated by the online tool (http://web.expasy.org/compute.pi/). All the precursors protein of differentially expressed peptides as one group were imported into for GO (http://www.geneontology.org/) (50) and pathway analysis (https://www.kegg.jp/) for predicting potential functions. The online tools UniProt Database (http://www.uniprot.org/) and Pfam (http://pfam.xfam.org/) were adopted to explore if the peptides' sequences were positioned in the conserved structural domains or regions of their precursors. The Open Targets Platform database (www.targetvalidation.org/) was adopted to investigate precursors associated with diabetes and obesity as previously reported (24). For visualization, clustergram and volcano plot graphs in this study were drawn with R language (http://www.r-project.org/). For determination of differentially expressed peptides, fold change was computed as the average values of biological duplication (*n* = 4).

### Synthetic Peptides

All the peptides used in this study were custom-synthesized and HPLC-purified by Science Peptide Biological Technology Co., Ltd. (Shanghai, China) through the solid-phase method as described reported (51). The purity in 95% for each peptide was confirmed by HPLC-MS method. All the used peptides were stored in lyophilization at−20°C until dissolved with sterile water immediately for treatment with cells *in vitro*.

### Cell Culture and Peptide Treatment

C2C12 cells, purchased from Cell Bank of the Chinese Academy of Sciences (Shanghai, China), were maintained in DMEM (Gibco, Carlsbad, CA, USA) supplemented with 10% Fetal Bovine Serum (Gibco) and 1% penicillin–streptomycin (Keygen Biotech, Nanjing, China) at 37°C with 5% CO2. On the fourth day of cell differentiation, the C2C12 cells were pre-treated with synthesized peptides or solvent for 48 h at the same concentration of 50 μM, and then starved for 24 h with serum-free L-DMEM (Gibco). Subsequently, myotubes were incubated in L-DMEM in the presence or absence of 100 nM of insulin for 30 min. At the indicated time, the cells were collected for the following analysis.

### Western Blot Analysis and Antibodies

At the indicated time, C2C12 myotubes were lysed in Radio Immunoprecipitation Assay (RIPA) lysis buffer containing protease and phosphatase inhibitors (Roche, Mannheim, Germany). Protein concentrations were measured with the BCA Protein Assay Kit (Thermo Fisher Scientific, Rockford, USA). Proteins were loaded and separated by 8%-10% (v/v) SDS-PAGE, transferred to polyvinylidene fluoride (PVDF) membrane and blocked with 5% milk. The membrane was incubated with the determined primary antibodies, respectively overnight at 4 °C as follows: Insulin receptor substrate 1 (Irs-1) (1:1000 in dilution, Cat No: 2382; Cell Signaling Technology, Danvers, MA, USA), Phospho-Irs-1(ser307) (1:1000 in dilution, Cat No: 2381; CST), RAC-alpha serine/threonine-protein kinase (Akt)(1:1000 in dilution, Cat No: 4685; CST), Phospho-Akt (Ser473)(1:1000 in dilution, Cat No: 4060; CST), Pgc1α(1:1000 in dilution, Cat No: ab54481; Abcam, Cambridge, UK), Tublin (1:1000 in dilution, Cat No: 10094-1-AP; Proteintech, Rosemont, USA). Then the membrane was incubated with goat anti-rabbit HRP secondary antibody (1:5000 in dilution, CAT: BL003A; Biosharp, Hefei, China). Proteins bands were visualized using a chemiluminescence kit and analyzed using Image J software.

### Reverse Transcription and Real-Time Quantitative PCR

Total RNA was isolated using trizol reagent (Life Technologies, Carlsbad, CA, USA). And the cDNA was synthesized by RevertAid First Strand cDNA Synthesis Kit (Thermo Fisher Scientific, Waltham, MA, USA). Gene expression was determined by real-time quantitative PCR (ABI ViiA7, Applied Biosystems, Foster City, California, USA) using the SYBR Green array. The relative gene expression was analyzed based on the 2^−ΔΔ^CT method with normalization of the data to PPIA. The primers used for the real-time quantitative PCR were listed in Table S1.

### Statistical Analysis

Data were analyzed with GraphPad Prism 7 (San Diego, CA, USA), and the results were shown as the mean ± standard deviation (SD). Peptides with a fold change larger than 1.5 or <0.67 with a Student's *t*-test *p*-value <0.05 were selected as differently expressed peptides.

## Results

### Identification of Secreted Peptides From Cultured *db/db* Skeletal Muscle Tissues

**Figure 1:**
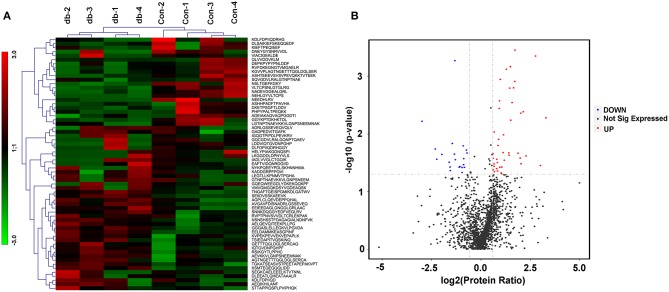
Differentially secreted peptides from the cultured skeletal muscle tissues from *db/db* and Control mice. **(A)** Heatmaps highlight the peptides intensity patterns differentially secreted from the medium supernatants of cultured skeletal muscle tissues from control (con) vs. *db/db* (db) groups, *n* = 4 per group. **(B)** Volcano plot after two-sample *t* test for peptides detected in medium supernatants derived from the cultured skeletal muscle from control and *db/db* mice. Red color indicates peptides we defined as up-regulated peptides and blue indicates down-regulated peptides.

Considering skeletal muscle tissues as an important secretory organ, which communicate with other organs though the secreted proteins, miRNAs, metabolites and others, we were interested in identifying peptides secreted from skeletal muscle tissues under the pathophysiology of metabolic diseases such as diabetes and obesity. LDH and IL-6 release were evaluated in the supernatant from skeletal muscle explants isolated from the control mice, which partly reflected the signs of tissue damage along the incubation period. LDH content of culture medium was assessed as an indicator of cell lysis at 0 h~24 h, 24 h~48 h and 48 h~72 h; no significant increased in LDH occurred from 48 h to 72 h (not shown in the manuscript). Secretion of IL-6 remained stable from 24 h to 48 h in culture by ELISA (not shown in the manuscript). Therefore, we chose the 24 h~48 h culture as an optimal time point. The protein composition and integrity of the secreted samples were evaluated by SDS-PAGE with silver staining in Figure S1. After validation of skeletal muscle culture system and samples assessment, peptides from four different culture dishes per group (control and db/db mice) were individually extracted from supernatants and analyzed via label-free mass spectrometry based quantification. To gain more insights into biological differences between control and db/db mice skeletal muscle, we employed LC-MS/MS technology and identified 3384 peptides, of which 2664 peptides showed valid quantitative values in the detection. A total of 63 peptides were identified differentially secreted, of which 42 peptides were up-regulated (fold change > 1.5, *P* < 0.05) and 21 peptides were down-regulated (fold change < 0.67, *P* < 0.05) in medium supernatants from insulin resistant-skeletal muscle tissues. Visualization methods such as hierarchical clustering and volcano plot showed distinct peptides secretion profiles as shown in Figures 1A,B. The differentially secreted peptides were summarized in Table 1.

**Table 1:** Differentially expressed peptides secreted from the medium supernatants of cultured skeletal muscle tissues from *db/db* and control mice.

**Peptide sequence**	**Protein ID**	**Protein name**	**Mr(KDa)**	**Fold change**	***P*-value**
**Up-regulated peptides**					
AVGAVFDISNADRLGSSEVEQ	P07310	KCRM	2163	9.728	0.005
TNGAFTGEISPGMIKDLGATWV	P17751	TPIS	2264	8.019	0.035
IADLVVGLCTGQIK	P21550	ENOB	1486	6.77	0.000
NYKPQEEYPDLSKHNNHMA	P07310	KCRM	2315	4.843	0.024
EEIEEDAGLGNGGLGRLAAC	Q9WUB3	PYGM	2030	4.411	0.004
GQCGDVLRALGQNPTQAEV	P09542	MYL3	2013	4.077	0.033
AGPLCLQEVDEPPQHAL	P70296	PEBP1	1873	4.038	0.004
EAFTVIDQNRDGIID	P97457	MLRS	1705	3.947	0.026
DLFDPIIQDRHGGY	P07310	KCRM	1645	3.668	0.022
KDLFDPIIQD	P07310	KCRM	1203	3.282	0.000
LDDVIQTGVDNPGHP	P07310	KCRM	1576	3.265	0.001
SEIIDVSSKAEEVK	A2ASS6	TITIN	1533	3.134	0.002
VIACIGEKLDE	P17751	TPIS	1246	2.976	0.003
GTNPTNAEVKKVLGNPSNEEM	Q545G5	MYL1	2180	2.838	0.006
ADRLGSSEVEQVQLV	P07310	KCRM	1629	2.804	0.001
IGQGTPIPDLPEVKRV	A2AQA9	NEB	1719	2.739	0.002
STTAPPIQSPLPVIPHQK	E9PYJ9	LDB3	1910	2.685	0.023
KADDGRPFPQVI	P05064	ALDOA	1342	2.644	0.021
KSMTEQEQQQLIDD	P07310	KCRM	1692	2.442	0.001
GGGASLELLEGKVLPGVDA	P09411	PGK1	1781	2.264	0.028
LEGTLLKPNMVTPGHA	P05064	ALDOA	1677	2.208	0.010
VMVGMGQKDSYVGDEAQSK	P68134	ACTS	2028	2.168	0.021
DLEEATLQHEATAAALR	Q02566	MYH6	1838	2.07	0.038
AELQEVQITEEKPLLPG	Q9QYG0	NDRG2	1935	2.043	0.048
SEGKCAELEEELKTVTNNL	P58771	TPM1	2163	2.038	0.049
RSIKGYTLPPHC	P07310	KCRM	1428	1.967	0.001
NSNSHSSTFDAGAGIALNDNFVK	P16858	G3P	2365	1.935	0.038
TGKATSEASVSTPEETAPEPAKVPT	P70402	MYBPH	2484	1.919	0.003
DNEYGYSNRVVDL	P16858	G3P	1543	1.898	0.004
EELDAMMKEASGPINF	P97457	MLRS	1781	1.853	0.012
SNNKDQGGYEDFVEGLRV	Q545G5	MYL1	2026	1.816	0.016
AEQIKHILANF	P63028	TCTP	1283	1.787	0.044
GADPEDVITGAFK	P97457	MLRS	1319	1.782	0.031
TSIEDAPITVQSKINQ	A2AQA9	NEB	1743	1.739	0.028
LKGGDDLDPNYVLS	P07310	KCRM	1505	1.732	0.040
IQTGVDNPGHPF	Q6P8J7	KCRS	1281	1.600	0.045
AEVKKVLGNPSNEEMNAK	Q545G5	MYL1	1957	1.595	0.009
GQEQWEEGDLYDKEKQQKPF	E9Q8K5	TITIN	2481	1.586	0.036
AGTNGETTTQGLDGLSERCA	P05064	ALDOA	2037	1.582	0.033
RVPTPNVSVVDLTCRLEKPAK	P16858	G3P	2378	1.531	0.022
KVPEKPEVVEKVEPAPLK	F7CR78	F7CR78	2015	1.527	0.026
GETTTQGLDGLSERCAQ	P05064	ALDOA	1822	1.526	0.042
**Down-regulated peptides**					
KIEFTPEQIEEF	P09542	MYL3	1509	0.612	0.021
DLSAIKIEFSKEQQEDF	P05977	MYL1	2026	0.600	0.019
HELYPIAKGDNQSPI	P16015	CAH3	1681	0.559	0.038
NSLTGEFKGKY	P07310	KCRM	1243	0.545	0.027
DEPKPYPYPNLDDF	Q3V1D3	AMPD1	1709	0.543	0.034
ASHHPADFTPAVHA	Q91VB8	HBA-A1	1457	0.537	0.016
SQVGDVLRALGTNPTNAE	Q545G5	MYL1	1841	0.516	0.047
ADEIAKAQVAQPGGDTI	P70349	HINT1	1725	0.515	0.038
DKETPSGFTLDDV	P07310	KCRM	1423	0.478	0.037
LGTNPTNAEVKKVLGNPSNEEMNAK	Q545G5	MYL1	2654	0.467	0.038
PHPYPALTPEQKK	P05064	ALDOA	1505	0.407	0.028
QLVVDGVKLM	P07310	KCRM	1101	0.400	0.001
KDLFDPIIQDRHG	P07310	KCRM	1553	0.395	0.022
KGVVPLAGTNGETTTQGLDGLSER	P05064	ALDOA	2399	0.367	0.015
RVFDKEGNGTVMGAELR	Q545G5	MYL1	1878	0.341	0.043
NADEVGGEALGRL	A8DUK4	HBB-BS	1300	0.339	0.037
NEHLGYVLTCPS	P07310	KCRM	1389	0.328	0.031
ASHTEEEVSVSVPEVQKKTVTEEK	E9Q8K5	TITIN	2669	0.253	0.023
NEEDHLRV	P07310	KCRM	1010	0.212	0.027
VLTCPSNLGTGLRG	P07310	KCRM	1444	0.201	0.018
GGYKPTDKHKTDL	P07310	KCRM	1459	0.127	0.006

### Charactrization of the Basic Feature of Differentially Secreted Peptides

**Figure 2:**
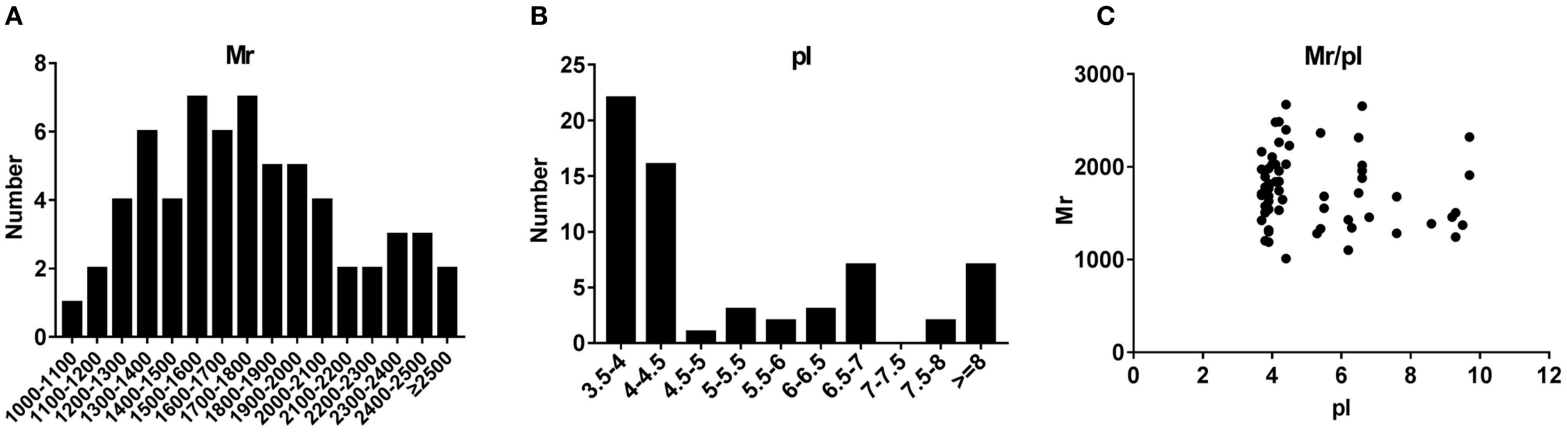
Charactrization of the basic feature of differentially secreted peptides. **(A)** Histogram displaying the Mr distribution of differentially secreted peptides from skeletal muscle peptidomics. **(B)** Histogram displaying the pI distribution of differentially secreted peptides from skeletal muscle peptidomics. **(C)** Scatter plot of Mr vs. pI.

To characterize the general feature of differentially secreted peptides, we further analyzed the relative molecular mass (Mr), isoelectric point (pI) and distribution of Mr vs. pI. The results indicated that the Mr varied from 1011.1 Da to 2670.9 Da with 70% between 1,300 and 2,100 Da (Figure 2A), and pI varied from 3.7 to 9.7, with 51% between 4 and 7 (Figure 2B). Additionally, distribution of pI vs. Mr could divide these peptides into three groups around pI4, pI6 and pI10 (Figure 2C).

### Comprehensive Functional Assessment and Intercellular Location of Precursor Proteins of These Differential Peptides

**Figure 3:**
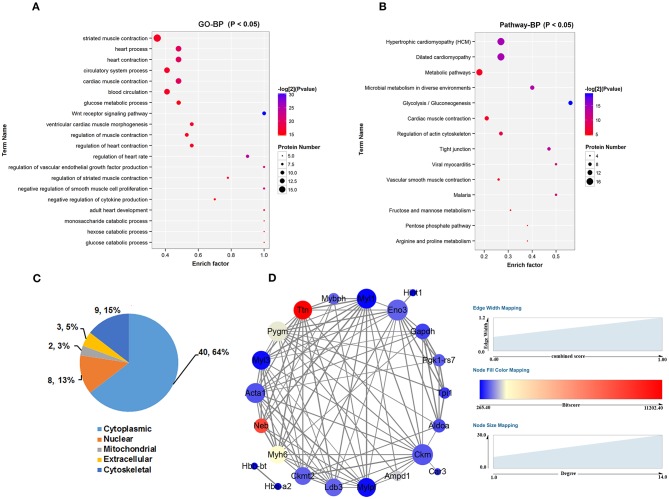
Comprehensive location and functional assessment of precursor proteins of these differential peptides. **(A)** GO analysis of precursor proteins and TOP 20 GO terms in the biological process categories. **(B)** Canonical pathways analysis of precursor proteins and TOP 14 pathways terms in the biological process categories. **(C)** Distribution of intercellular location of precursor proteins. **(D)** Search tool for the retrieval of interacting genes/proteins (STRING) was used to construct a graphical network for describing the interaction between these precursor proteins corresponding to the identified peptides. The size of node represents the number of interacting proteins and the color of node represents the bitscore of matching results from STRING database. The thicker the line (edge), the higher the reliability (evaluated by combined score >0.4).

To make comprehensive functional assessment of these differential peptides, we consider all the precursor proteins as one group and performed GO and Pathways analysis of them. First of all, the precursor proteins of these differential peptides identified from the cultured control and *db/db* skeletal muscle were classified using Gene Ontology categories, which revealed the majority of proteins were associated with striated muscle contraction and glucose metabolic process (Figure 3A). The top 20 GO terms were listed in Figure 3A. The precursor proteins annotated involved in these GO terms were presented in Table S2. Subsequent pathway analysis in the KEGG database revealed a significant enrichment in metabolic pathway and glycolysis/gluconeogenesis process (Figure 3B). The top 14 pathway terms were listed in Figure 3B. The precursor proteins annotated involved in these pathway terms were presented in Table S3. We further discriminated the up- and down-regulated peptides and performed GO and Pathways analysis of these two groups of precursor proteins. This analysis may bring overlapping terms such as phosphorylation, phosphagen metabolic process and phosphorus metabolic process in up- or down-regulated peptides, and a smaller number of terms in Pathway analysis (deriving up-regulated peptides). These analysis were presented in Figure S2. Additionally, the major intracellular locations of the precursor proteins were considered from literature sources, illustrating that 64% proteins were cytoplasmic and 15% proteins were cytoskeletal seen in Figure 3C. Based on above analysis, we found that a vast of precursor proteins were assigned to glucose metabolic process include ALDO A, triosephosphate isomerase (TPIS), beta-enolase (ENOB), glycogen phosphorylase, muscle form (PYGM), glyceraldehyde-3-phosphate dehydrogenase (GAPDH) and phosphoglycerate kinase 1(PGK1), specifically assigned to glycolysis/gluconeogenesis process. Another kind of proteins enriched in regulation of striated muscle contraction terms (GO) and regulation of actin cytoskeleton (Pathway) were myosin regulatory light chain 2, skeletal muscle isoform(MLRS), myosin heavy chain 6 (MYH6), myosin heavy chain 7 (MYH7), myosin-binding protein H (MYBPH) and myosin light chain 3 (MYL3),both belonging to sarcomeric proteins. Subsequently, we used search tool for the retrieval of interacting genes/proteins (STRING) to construct a graphical network for describing the interaction between these precursor proteins corresponding to the identified peptides in Figure 3D. Based on protein-protein interaction and co-occurrence in KEGG pathways and literature mining, this network was constructed from 25 proteins that matched to the STRING database and the matching results were listed in Table S4. As shown from this network, 22 nodes representing precursor protein constituted the interaction diagram, which could form 93 reliable a-to-b interaction relationship by combined score (more than 0.4) as shown in Table S5.

### Cleavage Pattern of Differentially Secreted Peptides

**Figure 4:**
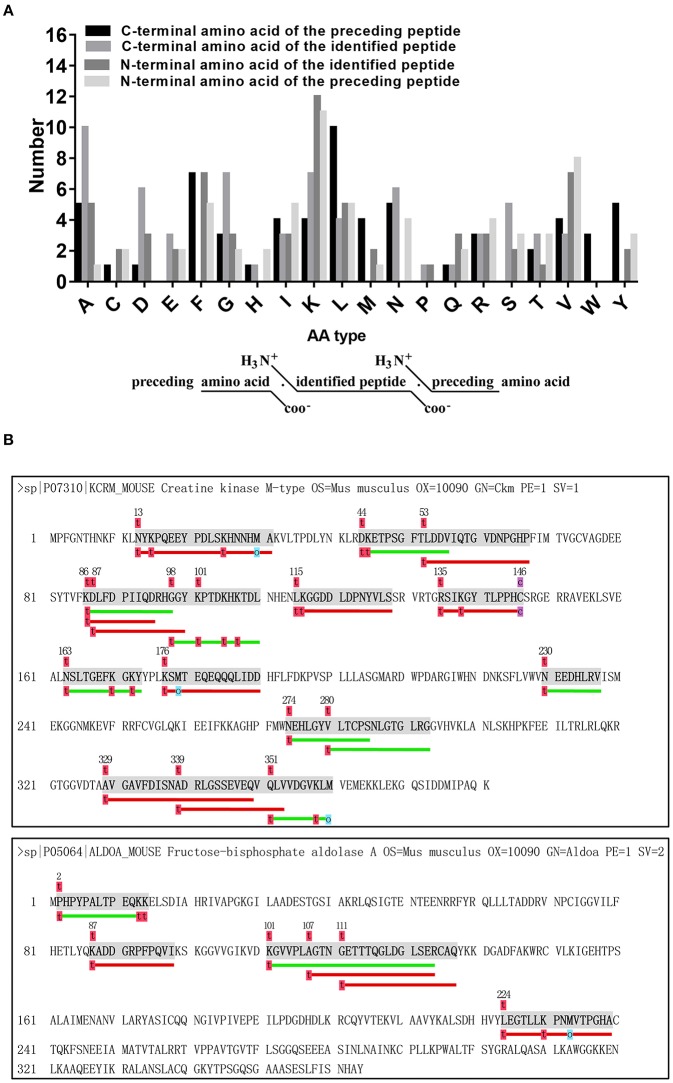
Analysis of cleavage pattern of differentially secreted peptides. **(A)** Distribution of the four cleavage sites in the identified differentially secreted peptides. **(B)** Peptides derived from the same precursor protein KCRM or ALDOA.

It is widely postulated that most peptides can be attributed mainly to the proteolytic enzymes as well as their type and level (cleavage specificity and activity), which can differ during disease (52–54). Briefly, the N-terminal pre-cleavage site, N terminus, C terminus, and C-terminal post-cleavage site of the identified peptides were commonly used to investigate the nature of proteolytic enzymes within serum (55) or tissues (56). Thus, we analyzed the distribution of peptide cleavage site and found that Lysine (K) was the most frequent cleavage site of N-terminal amino acid of identified peptides and Alanine (A) was the most frequent cleavage site of C-terminal amino acid of identified peptides. Lysine (K) was the most frequent cleavage site of N-terminal amino acid of preceding peptides, while Leucine (L) was the commonest C-terminal amino acid of preceding peptides (Figure 4A). These data indicate that the pattern of cleavage sites may represent the specificity of cleavage and activity of proteolytic enzymes under the diabetic condition. Additionally, we also tried to align peptide sequences on the same precursor sequence to construct a “peptide alignment map”. Searched from our samples, the largest number of identified peptides (*n* = 1 7) (Figure 4B) originated from KCRM and the second largest number of identified peptides (*n* = 6) came from ALDO A (Figure 4B), elucidating that these peptides were easily cleaved by certain kind of endogenous enzymes.

### Putative Bioactive Peptides Associated With Diabetes and Obesity

To check for specific domain structures or patterns in the identified peptides comparison with its precursor proteins, we retrieved domain information from the UniProt and Pfam database. In order to validate that if the peptides exerted important roles in the related metabolic diseases, we adopted the Open Targets Platform database to investigate protein precursors. In our searching results, most peptides (*n* = 44) from the identified pepetides (*n* = 63) were located in the functional domains (Table 2). Additionally, 27 out of 44 peptides located in the functional domain were predicted closely related with both obesity and diabetes (Table 3). These observations will encourage us to further investigate properties of the putative secreted peptides in skeletal muscle under the diabetic state.

**Table 2:** Differentially secreted peptides located in functional domain or region based on Uniprot or Pfam database.

**Peptide sequence**	**Protein**	**Location**	**Domain**	**Description**
**Up-regualted peptides**				
AVGAVFDISNADRLGSSEVEQ	KCRM	329–349	125–367	Phosphagen kinase C-terminal
TNGAFTGEISPGMIKDLGATWV	TPIS	121–142	57–295	TIM
IADLVVGLCTGQIK	ENOB	381–394	142–432	Enolase C
NYKPQEEYPDLSKHNNHMA	KCRM	13–31	11–98	Phosphagen kinase N-terminal
EEIEEDAGLGNGGLGRLAAC	PYGM	124–143	113–828	Phosphorylase
GQCGDVLRALGQNPTQAEV	MYL3	83–101	58–95	EF-hand 1
EAFTVIDQNRDGIID	MLRS	32–46	25–60	EF-hand 1
DLFDPIIQDRHGGY	KCRM	87–100	11–98	Phosphagen kinase N-terminal
KDLFDPIIQD	KCRM	86–95	11–98	Phosphagen kinase N-terminal
LDDVIQTGVDNPGHP	KCRM	53–67	11–98	Phosphagen kinase N-terminal
VIACIGEKLDE	TPIS	174–184	57–295	TIM
GTNPTNAEVKKVLGNPSNEEM	MYL1	39–59	6–41	EF-hand
ADRLGSSEVEQVQLV	KCRM	339–353	125–367	Phosphagen kinase C-terminal
KADDGRPFPQVI	ALDOA	87–98	15–364	Glycolytic
KSMTEQEQQQLIDD	KCRM	177–190	125–367	Phosphagen kinase C-terminal
GGGASLELLEGKVLPGVDA	PGK1	395–413	9–406	PGK
LEGTLLKPNMVTPGHA	ALDOA	224–239	15–364	Glycolytic
VMVGMGQKDSYVGDEAQSK	ACTS	45–63	4–377	Actin
DLEEATLQHEATAAALR	MYH6	1,179–1,195	845–1,926	Myosin_tail_1
SEGKCAELEEELKTVTNNL	TPM1	186–204	1–284	Coiled coili
RSIKGYTLPPHC	KCRM	135–146	125–367	Phosphagen kinase C-terminal
SNNKDQGGYEDFVEGLRV	MYL1	77–94	83–118	EF-hand
AEQIKHILANF	TCTP	119–129	1–172	TCTP
GADPEDVITGAFK	MLRS	93–105	95–130	EF-hand 2
LKGGDDLDPNYVLS	KCRM	115–128	125–367	Phosphagen kinase C-terminal
GQEQWEEGDLYDKEKQQKPF	TITIN	1,691–1,710	1,709–1,799	Ig-like
AGTNGETTTQGLDGLSERCA	ALDOA	117–136	15–364	Glycolytic
RVPTPNVSVVDLTCRLEKPAK	GAPDH	232–252	243–248	[IL]-x-C-x-x-[DE] motif
GETTTQGLDGLSERCAQ	ALDOA	121–137	15–364	Glycolytic
**Down-regualted peptides**				
KIEFTPEQIEEF	MYL3	52–63	58–95	EF-hand 1
DLSAIKIEFSKEQQEDF	MYL1	33–49	44–79	EF-hand 1
HELYPIAKGDNQSPI	CAH3	17–31	3–259	Alpha-carbonic anhydrase
NSLTGEFKGKY	KCRM	163–173	125–367	Phosphagen kinase C-terminal
ASHHPADFTPAVHA	HBA-A1	111–124	3–142	GLOBIN
DKETPSGFTLDDV	KCRM	44–56	11–98	Phosphagen kinase N-terminal
LGTNPTNAEVKKVLGNPSNEEMNAK	MYL1	76–100	83–118	EF-hand
QLVVDGVKLM	KCRM	351–360	125–367	Phosphagen kinase C-terminal
KDLFDPIIQDRHG	KCRM	86–98	11–98	Phosphagen kinase N-terminal
KGVVPLAGTNGETTTQGLDGLSER	ALDOA	111–134	15–364	Glycolytic
RVFDKEGNGTVMGAELR	MYL1	147–163	133–150	EF-hand
NADEVGGEALGRL	HBB-BS	20–32	2–147	GLOBIN
NEHLGYVLTCPS	KCRM	274–285	125–367	Phosphagen kinase C-terminal
NEEDHLRV	KCRM	230–237	125–367	Phosphagen kinase C-terminal
VLTCPSNLGTGLRG	KCRM	280–293	125–367	Phosphagen kinase C-terminal

**Table 3:** Protein precursors of peptides which are both associated with obesity and diabtetes (Score >0).

**Protein name**	**Description**	**Peptide number**	**Association score with obesity^#^**	**Association score with diabetes mellitus^#^**
TPI1	Triosephosphate isomerase	2	0.012	0.012
KCRM	Creatine kinase M-type	17	0.022	0.017
TTN	Titin	1	0.026	0.063
GAPDH	Glyceraldehyde-3-phosphate dehydrogenase	1	0.071	0.117
HBA-A1	Alpha globin 1	1	0.014	0.038
ENOB	Beta-enolase	1	0.040	0.048
CAH3	Carbonic anhydrase 3	1	0.074	0.094
TCTP	Translationally-controlled tumor protein	1	0.028	0.040
HBB-BS	Beta-globin	1	0.008	0.020
ACTS	Actin, alpha skeletal muscle	1	0.006	0.188

# *The association score come from open targets platform database*.

### The Effect of Candidated Peptides (P1-P7) on Insulin and Mitochondrial-Related Pathways in Skeletal Muscle Cells

**Figure 5:**
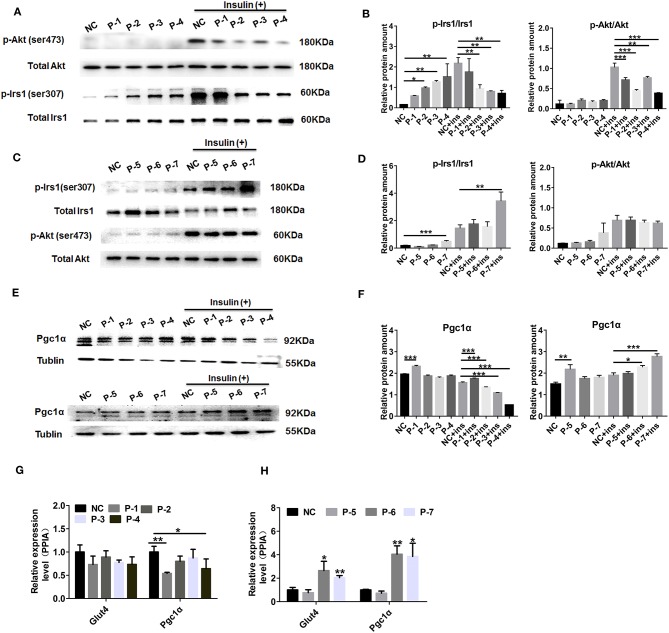
Effect of the candidate peptides on insulin and mitochondrial-related pathways. The differentiated C2C12 motes were treated with the candidate peptides (50 uM) named P1–P7 groups or solvent termed untreated groups. After incubation with or without 100 nM insulin for 30 min, cell lysates were analyzed by Western blot. **(A,C)** Total protein and phosphorylation of Irs1 at ser307 (p-Irs1) and Akt at ser473 (p-Akt). **(B,D)** Quantification of the protein levels of p-Irs1 (ser307) and p-Akt (ser473) relative to total protein, respectively. **(E)** Mitochondrial-related protein Pgc1α was detected and Tublin was used as internal control. **(F)** Quantification of the protein levels of Pgc1α relative to Tublin. Values are the means ± SD of three separate experiments. **(G,H)** Real-time quantitative PCR detection of the mRNA levels of Glut4 and Pgc1 relative to internal control PPIA, respectively. Values are the means ± SD of three separate experiments. ^*^P < 0.05; ^**^P < 0.01; ^***^P < 0.001.

To explicit the putative function of differentially secreted peptides, 7 differentially secreted peptides, which had already been annotated derived from authentic protein from Uniprot database, located in the functional motifs and showed relatively high abundance from MS detection, were chosen for further analysis. The up-regulated peptides were AVGAVFDISNADRLGSSEVEQ (KCRM/P07310), ADRLGSSEVEQVQLV (KCRM/P07310), GADPEDVITGAFK (MLRS/P97457), RVPTPNVSVVDLTCRLEKPAK (GAPDH/P16858), whereas the down-regulated peptides were DKETPSGFTLDDV (KCRM/P07310), NEHLGYVLTCPS (KCRM/P07310), VLTCPSNLGTGLRG (KCRM/P07310) as listed in Table S6. Sequentially, we termed these candidated peptides P1-P7 in order. To use C2C12 myotubes as cell models *in vitro*, the results of changes in inuslin signaling revealed that up-regulated peptides P2-P4 addition could both significantly decrease the phosphorylation of Irs1 (Ser-307) and Akt (Ser-473) under insulin stimulation (100 nM, 30 min) as shown in Figures 5A,B, in spite of bringing a modestly up-regulation of p-Irs1 (Ser-307) at the basal status. Moreover, mitochondrial-related marker protein Pgc1α was significantly attenuated in the insulin-stimulated C2C12 cells by up-regulated peptides P1-P4 as shown in Figures 5E,F. Among these down-regulated peptides, only peptide 7 presented a promotion on p-Akt (Ser-473) expression level both under the basal and insulin stimulation status as shown in Figures 5C,D. And these up-regulated peptide 1-4 could decrease Pgc1α protein level and down-regulated peptide 6 and 7 administration enhanced Pgc1α protein level by the stimulation of insulin presented in Figures 5E,F. We further evaluated Gut 4 (a key gene for glucose transport) and Pgc1α mRNA levels by peptides treatment upon insulin stimulation. The results also indicated that Peptide 3 and 4 modestly decreased Pgc1αexpression at mRNA levels seen in Figure 5G, while Peptide 6 and 7 significantly up-regulated both Glut4 and Pgc1α expression at mRNA levels seen in Figure 5H. These observations revealed that these candidated peptides (P1-P7) may affect the expressions of insulin signaling or mitochondrial-related genes in skeletal muscle cells.

## Discussion

Emerging as a widely known secrete organ, skeletal muscle tissues have been extensively discussed using a wide range of comparative and quantitative proteomic methods (57). Proteomic scannings of the medium supernatant from skeletal muscle cells have already afford approaches to identify a large number of secreted proteins (58–63). Particularly, one such research reported the alteration of insulin effect on the secretome profile of skeletal muscle cells, revealing the changes of protein levels secreted from skeletal muscle during activation of insulin signaling pathway (58). A recent study also generated a comprehensive secretome analysis of skeletal muscle cells under palmitic acid-induced insulin resistance (61). In addition to these proteins, many other muscle-secreted compounds have been also come to light, including exosome (64, 65), metabolites (66), and miRNAs (67). These studies have greatly expanded our knowledge of skeletal muscle secretome and might afford possibilities for exploring novel molecular targets in the maintenance of skeletal muscle physiology and even whole-body metabolism. However, to date few studies has focused on the peptides present in either skeletal muscle tissues or cells. Therefore, we attempted to comprehensively profile peptides that may play roles in regulating insulin sensitivity and offer enormous promise for exploring molecular mechanisms underlying insulin resistance.

In present study, a total of 63 peptides were differentially secreted in medium supernatants from cultured skeletal muscle tissues of *db/db* mice, of which 42 peptides were up-regulated (fold change > 1.5, *P* < 0.05) and 21 were down-regulated (fold change < 0.67, *P* < 0.05) shown in Figures 1A,B. The differences of dysregulation peptides may indeed reflect the changes between control and insulin-resistant mice in some extent. However, *db/db* mouse is a well-established leptin receptor-deficient animal model (68, 69). Despite no studies revealed the association between leptin receptor deficiency and peptide dysregulation/protein degradation so far, the possible effect by this mutation deserves to be taken into account and other non-genetic mice models could be adopted in the future studies. Based on our previous study (70), 10-kDa filters method was used to extract peptides in order to intercept proteins secreted by skeletal muscle to the conditional media. Characterization of the basic feature (Mr and pI) of these identified peptides (Figures 2A,B) only reflected the differences of distribution in the diabetic skeletal muscle tissues, but also proved the reliability of the used peptide extraction method.

Subsequently, GO and Pathway analysis revealed that the precursors protein of these peptides were mostly involved in muscle contraction and metabolism processes (Figures 3A,B). An interesting finding from our study was that most of the differentially identified peptides derived from cytosolic, cytoskeletal and mitochondrial proteins (Figures 3A,B), which differed from the secretory pathway peptides (71). This observation was also demonstrated by other peptidomic studies. From Steven W. Taylor group' results, several identified peptides in the human islet cultures were derived from intracellular and cytoskeletal proteins such as microtubule-associated protein 4 and ubiquitin, which may result from a greater level of cellular stress (34). Similarly, another peptidomic anaylsis of brain slices cultures and media also pointed that vast majority of secreted peptides arose from intracellular proteins (72). There does also exist some evidence that these identified N- or C-terminus protein yielding peptides, rather than internal fragments, raised the possibility that they are produced by selective processing rather than protein degradation (73). Taken together with previous researches, the current results show us meaningful hints that these intracellular peptides may be secreted via non-classical mechanisms. Actually, another important origin of peptides is proteolytic degradation processes in body fluid under the physiological or pathologically processes. Most regulatory peptides were efficiently degraded by plasma enzymes once secreted into the bloodstream, which exerted anti-diabetic therapeutic function (74, 75) or were identified as disease markers (76). Recently, Federico Aletti et al. (77) used protease activity detection and specific enzymes analysis to explain a large presence of circulating peptides under hemorrhagic shock, which gave us a possible way to evaluate peptides origin. Thus, a fundamental validation is whether the proteins-derived peptides are actually secreted from skeletal muscle cells per se or proteolytic degradation and are of biologically active.

Among the differentially secreted peptides from cultured skeletal muscle tissues of *db/db* mice, we found that three peptides derived from GAPDH, four peptides derived from ALDO A and one peptides derived from PYGM were up-regulated in the conditional medium from *db/db* groups as shown in Table 1. As described by Dustin S. Hittel et.al, a global proteomic survey of skeletal muscle revealed a statistically significant up-regulation in glycolytic enzymes GAPDH and ALDO A protein levels in obese/overweight patients (78). Another quantitative protein profile also identified a more abundant levels of GAPDH and PYGM in skeletal muscle from T2DM groups compared with the control groups (79). In addition to pointing the importance of the mitochondrial numbers and impairments under the insulin-resistant states (80–82), several studies noted that glycolytic capacity is higher in skeletal muscle of patients with T2DM or obesity (83). Notably, stronger changes of peptides derived from sarcomeric proteins such as myosin light chain 1 (MYL1) and MYL3 were also observed in the conditional medium from diabetic muscles as shown in Table 1. MYL1 and MYL3 are representive markers of fast-muscle and slow-muscle respectively, which were both regulated by insulin stimulation (79). And previous studies observed that property of T2DM individuals muscle was shifted to a fast-twitch glycolytic phenotype (84). In fact, deficiency in sarcomeric proteins in skeletal muscle also suggested their importance in skeletal muscle physiologic and pathological processes. On the whole, our results of increased glycolytic enzyme- or sarcomeric proteins- derived peptides secreted from insulin resistant skeletal muscle may support the hypothesis that altered glycolytic capacities or fiber types under the diabetic status contribute to this difference.

Till now, the putative function of these peptides are not clear. Therefore, we evaluated whether these peptides originated from functional domains of the corresponding precursor protein using the UniProt and Pfam database. In our searching results, most peptides (*n* = 44) from the identified peptides (*n* = 63) were located in the functional domains as listed in Table 2. Specifically, most peptides derived from functional enzymes were located in the enzymatic activity region, including TPI-derived peptides (121-TNGAFTGEISPGMIKDLGATWV-142, 174-VIACIGEKLDE-184), PGK-derived peptide (395-GGGASLELLEGKVLPGVDA-413), ALDOA-derived peptides (87-KADDGRPFPQVI-98, 224-LEGTLLKPNMVTPGHA-239, 117-AGTNGETTTQGLDGLSERCA-136, 121-GETTTQGLDGLSERCAQ-137), TCTP-derived peptide (119-AEQIKHILANF-129) and CAH3-derived peptide (17- HELYPIAKGDNQSPI-31). We also found another kind of peptides (*n* = 9) were located in the EF-hand domain from sarcomeric proteins (MYL1, MYL3 and MLRS). Generally, all EF-hand proteins display regulatory effect in two ways (85), calcium sensors for translating the signal to various responses and calcium buffers for controlling free Ca2^+^ ions level in the cytoplasm. On the other side, Ca2^+^ binding could induce a change of structural dynamics in the EF-hand motif, resulting in the activation or inactivation of target proteins (86, 87). Importantly, Ca2^+^ signal participates a variety of physiological processes in skeletal muscle, especially acting as second messengers for GLUT4 translocation mediated by contraction (88) and insulin treatment (89). Additionally, GAPDH-derived peptide (231- RVPTPNVSVVDLTCRLEKPAK−252) contained [IL]-x-C-x-x-[DE] motif (243-248), which has been reported as S-Nitrosylation modifying sites for affecting GAPDH enzymatic activity (90). Therefore, future efforts need to be established for investigating the potential role of peptides on insulin sensitive cells *in vitro* or whole-body metabolism *in vivo*.

The above analyses provided a possibility to evaluate the biological effects of these differentially secreted peptides. As widely reported, insulin resistance in skeletal muscle is tightly connected with the deficit in insulin signaling (91). Consequently, the role of phosphorylation of Irs1 and Akt in signaling pathways is very crucial in anti-hyperglycemia and insulin sensitivity (92). In this result, we found these up-regulated peptides 1–4 both exerted a significantly attenuated insulin action in C2C12 cells, evaluated by a decreased level of p-Irs1 (Ser-307) and p-Akt (Ser-473) seen in Figures 5A,B, whereas only one down-regulated peptide P7 could remarkably promote insulin signaling only via Irs1 signaling pathway seen in Figures 5C,D. Peroxisome proliferator-activated receptor γ coactiva-tor-1(Pgc-1), which displays a dominant role through tight modulation of mitochondrial biogenesis and respiration, has also been demonstrated to participate in skeletal muscle insulin signaling and metabolic homeostasis (93). The up-regulated peptides 1–4 also brought out a decreased protein level of Pgc-1α in C2C12 cells as shown in Figures 5E,F, and down-regulated peptide 6 and 7 administration also gave rise to the increased Pgc1α mRNA and protein level in Figures 5E,F,H. Taken together, these selected peptides secreted from *db/db* mice skeletal muscle presented a promotive or inhibitory effect on insulin and mitochondrial-related pathways in skeletal muscle cells by an autocrine manner. Notably, peptide 4 (231- RVPTPNVSVVDLTCRLEKPAK−252) derived from GAPDH displayed a most significant inhibitory effect toward these candidate peptides. However, the relationship between GAPDH-derived peptide and its precursor protein is to be determined. On the other hand, more methods need to be employed for the wider cell signaling screen and further research is required to explore the biologic function of skeletal muscle-secreted peptides on adipocytes or liver cells.

To our knowledge, no large-scale quantitative peptidomic analysis has been performed on skeletal muscle to elucidate secreted peptides profiles under the diabetic status. The present study identified and quantified changes with a label-free discovery using LC-MS/MS technology to construct a global secreted peptides picture. Further bioinformatics analysis of precursors comprehensively provided an atlas of peptides that may exist roles in regulating insulin sensitivity. This represented a new perspective toward exploring insulin resistance pathogenesis. Additionally, the detailed biological effects of these secreted peptides on skeletal muscle insulin resistance or cross-talk with other tissues remained to be elucidated in the future study.

## Data Availability Statement

The raw data supporting the conclusions of this manuscript will be made available by the authors, without undue reservation, to any qualified researcher.

## Ethics Statement

The protocol has been approved by the Institutional Animal Care and Use Committee of Nanjing medical university (Approval Number: IACUC-1812053).

## Author Contributions

YWu and MH performed experiments and interpreted results of experiments. YWa and YG prepared the figures. XC and PX analyzed the data. CJ and TZ participated discussion. YZ helped write the manuscript with providing assistance. LY conceived and designed experiments, provided funding to regents, and approved final version of manuscript. All authors read and approved the final manuscript.

### Conflict of Interest

The authors declare that the research was conducted in the absence of any commercial or financial relationships that could be construed as a potential conflict of interest.

## Supplementary Material

Figure S1 SDS-PAGE associated with silver staining of the medium supernatants from cultured skeletal muscle tissues (Con vs. *db* groups).

Figure S2 Functional assessment of precursor proteins deriving up- and down-regulated peptides, respectively. **(A,B)** GO analysis of precursor proteins deriving up- and down-regulated peptides, respectively. **(C,D)** Canonical pathways analysis of precursor proteins deriving up- and down-regulated peptides, respectively.

Table S1 Primers used in this study.

Table S2 GO terms corresponded to the precursor proteins relative to these differentially secreted peptides in skeletal muscle from *db/db* mice.

Table S3 Pathway terms corresponded to the precursor proteins relative to these differentially secreted peptides in skeletal muscle from *db/db* mice.

Table S4 The matching results of the precursor proteins from STRING database.

Table S5 Precursor proteins-to-precursor proteins interaction from STRING database.

Table S6 The information of candidate peptides for functional evaluation.

## Abbreviations

T2DM: type 2 diabetic mellitus
GO: gene ontology
KCRM: creatine kinase m-type
Aldo A: fructose-bisphosphate aldolase A
Mr: relative molecular mass
pI: isoelectric point
LC-MS/MS: Liquid chromatography tandem mass spectrometry
FA: formic acid
DDA: data dependent acquisition
FDR: false discovery rate
PSPEP: Proteomics System Performance Evaluation Pipeline
RIPA: Radio Immunoprecipitation Assay
PVDF: polyvinylidene fluoride
SDS: sodium dodecyl sulfate
PAGE: polyacrylamide gel electrophoresis
SD: standard deviation
TPIS: triosephosphate isomerase
ENOB: beta-enolase
PYGM: glycogen phosphorylase, muscle form
GAPDH: glyceraldehyde-3-phosphate dehydrogenase
PGK1: phosphoglycerate kinase 1
MLRS: myosin regulatory light chain 2, skeletal muscle isoform
MYH6: myosin heavy chain 6
MYH7: myosin heavy chain 7
MYBPH: myosin-binding protein H
MYL3: myosin light chain 3
MYL1; LDH: myosin light chain 1; lactate dehydrogenase.
