# Supplementary figures and images for: A Comparative Peptidomic Characterization of Cultured Skeletal Muscle Tissues Derived From db/db Mice
Source: Front Endocrinol (Lausanne). 2019 Oct 29;10:741. doi: 10.3389/fendo.2019.00741 (PMC6828820; doi:10.3389/fendo.2019.00741)

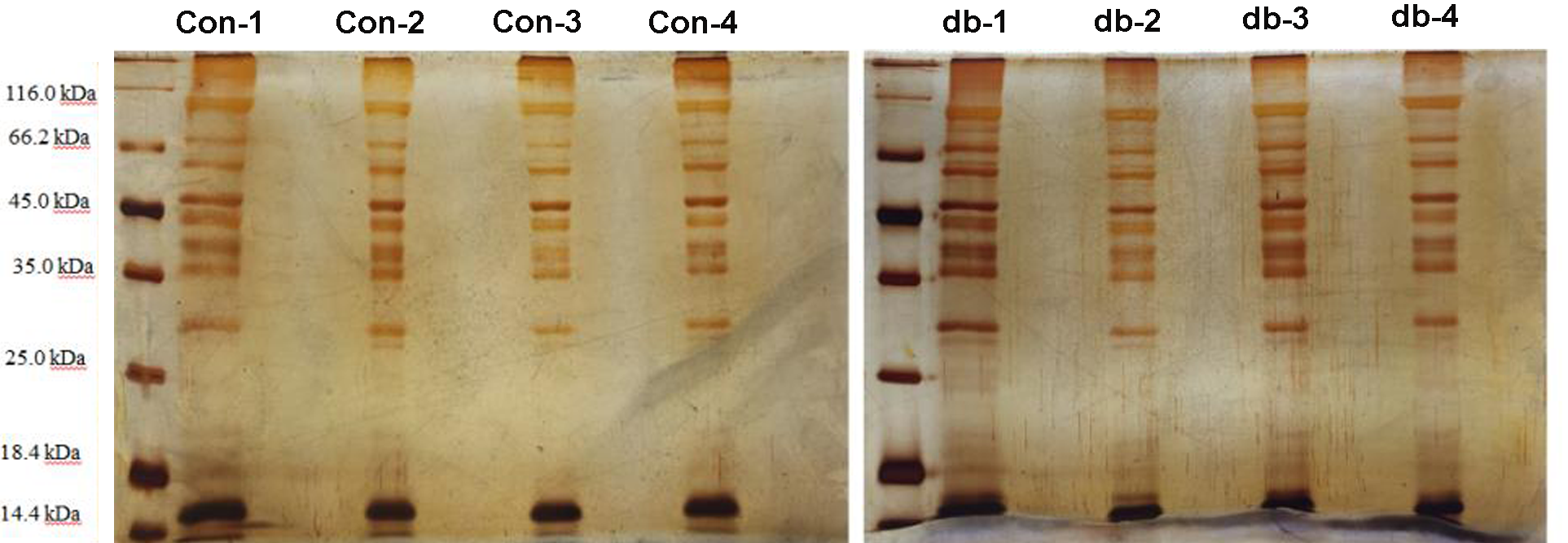

Supplement: Figure S1 — SDS-PAGE associated with silver staining of the medium supernatants from cultured skeletal muscle tissues (Con vs. db groups). [file Image_1.TIF]

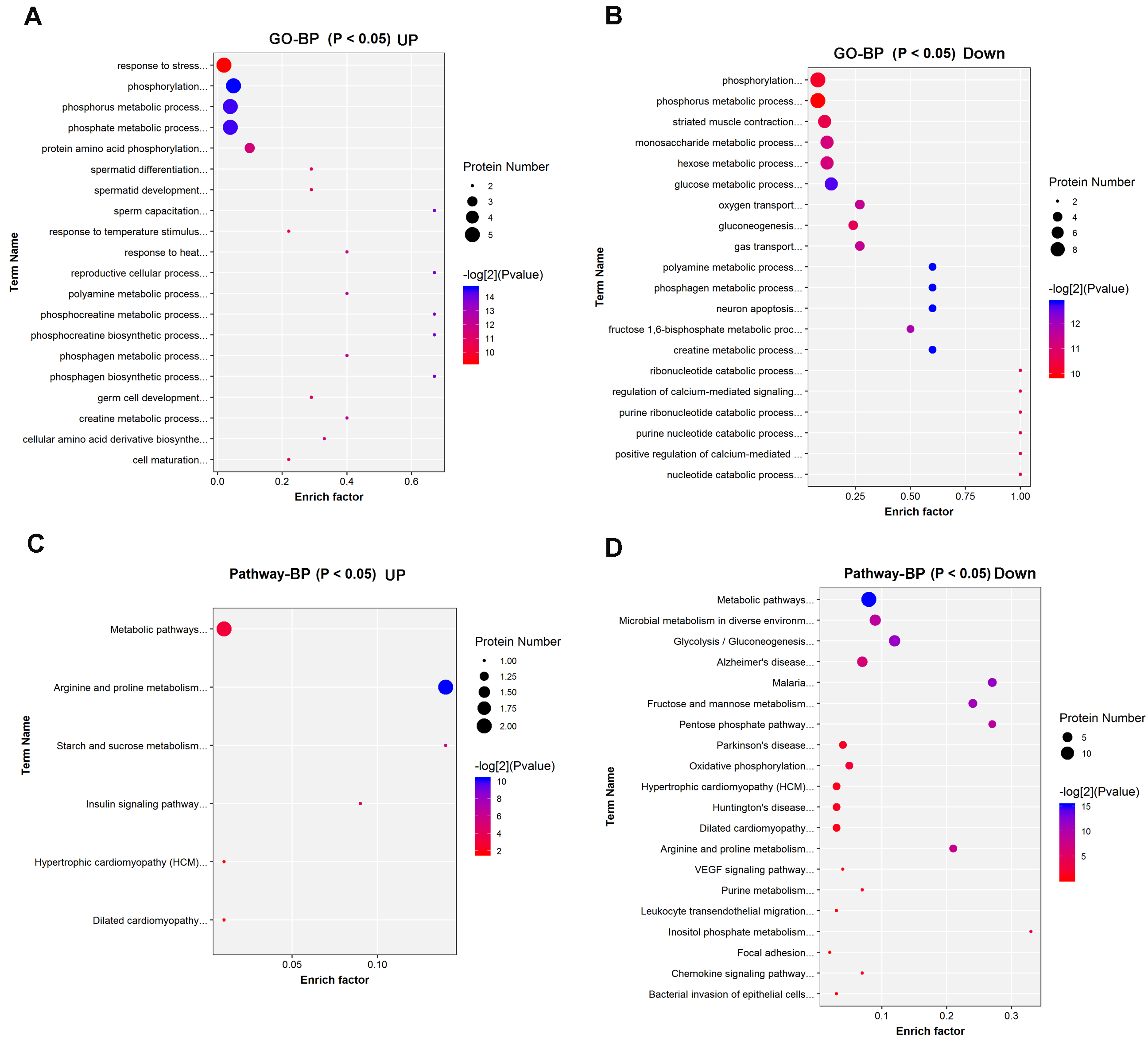

Supplement: Figure S2 — Functional assessment of precursor proteins deriving up- and down-regulated peptides, respectively. (A,B) GO analysis of precursor proteins deriving up- and down-regulated peptides, respectively. (C,D) Canonical pathways analysis of precursor proteins deriving up- and down-regulated peptides, respectively. [file Image_2.TIF]
